# Polydatin administration attenuates the severe sublesional bone loss in mice with chronic spinal cord injury

**DOI:** 10.18632/aging.204382

**Published:** 2022-11-15

**Authors:** Jiheng Zhan, Dan Luo, Bingde Zhao, Shudong Chen, Jiyao Luan, Junhua Luo, Yu Hou, Yonghui Hou, Wenke Xu, Wanying Yan, Ji Qi, Xing Li, Qing Zhang, Dingkun Lin

**Affiliations:** 1 Department of Orthopaedics, The Second Affiliated Hospital of Guangzhou University of Chinese Medicine, Guangzhou 510120, China; 2 Postdoctoral Workstation, Guangdong Provincial Hospital of Chinese Medicine, Guangzhou 510120, China; 3 Research Team on the Prevention and Treatment of Spinal Degenerative Disease, Guangdong Provincial Academy of Chinese Medical Sciences, Guangzhou 510006, China; 4 Postdoctoral Research Station, Chinese Academy of Chinese Medical Sciences, Beijing 100700, China; 5 Second Clinical College, Guangzhou University of Chinese Medicine, Guangzhou 510405, China; 6 Luoyang Orthopedic-Traumatological Hospital of Henan Province (Henan Provincial Orthopedic Hospital), Zhengzhou 450046, China; 7 National Quality Testing Center for Processed Food, Guangzhou Inspection and Testing Certification Group Company Limited, Guangzhou 511447, China; 8 Department of Spine, Wangjing Hospital of Chinese Academy of Chinese Medical Sciences, Beijing 100102, China

**Keywords:** polydatin, chronic spinal cord injury, bone loss, Wnt/β-catenin pathway

## Abstract

Background: Spinal cord injury (SCI) is often accompanied by rapid and extensive bone mineral loss below the lesion level, and there is currently no gold standard for treatment. Evidence suggests that polydatin (PLD) may help promote osteogenic differentiation and exhibit anti-osteoporotic activity. However, whether PLD could reverse substantial bone loss in SCI patients, especially those with protracted injury, and the underlying regulatory mechanism have not been investigated.

Study design: Male C57BL/6J mice were subjected to either contusion SCI or laminectomy at the T_8-9_ level. Eight weeks after SCI, PLD (40 mg/kg/day) or vehicle was administrated to the mice via the intragastric route for consecutive eight weeks. Blood was collected after the treatment regimen, and the tibiae and femora were removed. Bone marrow stromal cells were isolated from the long bones for *ex vivo* osteoblastogenesis and osteoclastogenesis assays.

Results: Chronic SCI led to a rapid and significant decrease in bone mineral density (BMD) of the distal femur and proximal tibia, resulting in structural deterioration of the bone tissues. Treatment with PLD largely restored BMD and bone structure. In addition, static histo-morphometric analysis revealed that PLD enhanced bone formation and inhibited bone resorption *in vivo*. PLD also promoted osteoblastogenesis and inhibited osteoclastogenesis *ex vivo*, which was accompanied by increased OPG/RANKL ratio, and reduced expression levels of CTR, TRAP, NFATc1 and c-Fos. However, PLD had no marked effect on serum 25(OH)D levels and VDR protein expression, although it did significantly lower serum and femoral malondialdehyde levels, inhibited expression level of matrix metallopeptidase 9 (MMP9), upregulated skeletal Wnt3a, Lrp5 and ctnnb1 mRNAs, and increased β-catenin protein expression.

Conclusions: PLD protected mice with chronic SCI against sublesional bone loss by modulating genes involved the differentiation and activity of osteoclasts and osteoblasts, abating oxidative stress and MMP activity, and restoring the Wnt/β-catenin signaling pathway.

## INTRODUCTION

Spinal cord injury (SCI) is a devastating neurological disease that can cause permanent disability and greatly lower the quality of life. In addition, significant sublesional bone loss may occur within several months to a few years after trauma [1]. The epiphyseal and metaphyseal trabeculae of the distal femurs and proximal tibiae are most frequently affected sites, and undergo maximum decrease in bone mass [2]. Long-term follow-up data suggests that the weekly bone loss rate may approach 1% throughout the acute to subacute post-injury period [1]. As a result, patients with SCI are at twice the risk of developing fragility fractures compared to the general population, and the risk increases over time [3, 4]. SCI-induced progressive bone loss is also correlated to a high incidence of osteoporotic fragility fractures, as well as increased disability, mortality and substantial health care costs [5].

However, the mechanisms underlying the initiation and progression of SCI-induced sublesional bone loss have not yet been elucidated. The generation and survival of osteoclasts, osteoblasts and osteocytes depend on the levels of reactive oxygen species (ROS) [6]. Excessive ROS production activates matrix metalloproteinases (MMPs), resulting in the degradation of bone matrix and increased bone resorption [7, 8]. Sustained oxidative stress and lower anti-oxidant activity are considered as the hallmarks of SCI and may also be involved in the progression of SCI-induced osteoporosis [9]. Vitamin D deficiency is a risk factor of post-SCI bone loss and fragility fractures [10]. Administration of Vitamin D analog for 6 months significantly improved bone mineral density (BMD) of lower limbs, and reduced urinary excretion of N-telopeptide in patients with chronic SCI [11]. In addition, bone loss after SCI is associated with undersupply of osteoblasts and oversupply of osteoclasts [12]. The Wnt/*β*-catenin signaling pathway is the major regulator of bone formation and resorption [13]. A recent study reported lower Wnt activity in the osteoblasts of the sublesional long bones after SCI [14]. Thus, therapeutic strategies that target these pathways may reverse the bone loss following SCI.

**Figure 1:**
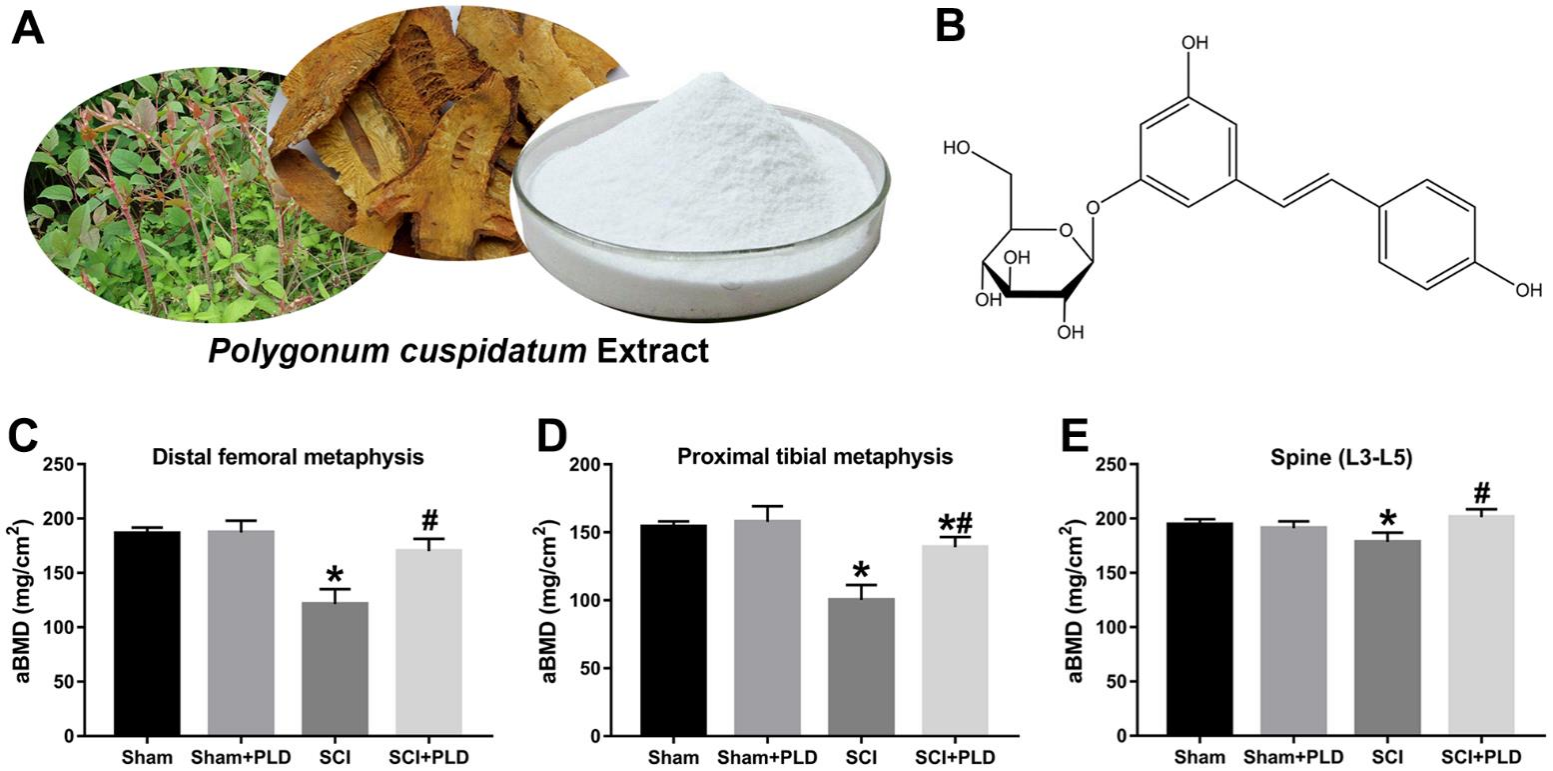
**PLD reverses the loss in BMD after chronic SCI.** (**A**) Representative images of *Polygonum cuspidatum,* giant knotweed, and polydatin (PLD). (**B**) The structure of PLD. Areal BMD acquired by DEXA scanning at (**C**) distal femoral metaphysic, (**D**) proximal tibial metaphysic, and (**E**) lumbar spine (L3-L5) in different groups. Data in panels represent as mean ± S.D.; n=8 in each group; *P < 0.05, vs. the Sham group; #*P* < 0.05, vs. the SCI group.

Polydatin (PLD, also named piceid or 3,5,4’-trihydroxystilbene-3-b-D-glucoside), the natural glycosylated precursor of resveratrol (RES), is a stilbenoid compound predominantly isolated from the dried rhizomes and roots of *Polygonum cuspidatum* (Figure 1A, 1B) [15]. In recent years, PLD has attracted considerable attention for its anti-oxidant, anti-inflammatory and anti-apoptotic activities. In addition, PLD exhibited neuroprotective effects in the mouse model of SCI [16, 17]. Although RES alleviated sublesional cancellous bone loss in rats with complete spinal transection, its clinical applications are limited due to low bioavailability and rapid metabolism [18]. PLD prevented bone loss in ovariectomized (OVX) animals, which is an established model of postmenopausal osteoporosis [19, 20]. Moreover, PLD can promote the proliferation and osteogenic differentiation of human bone marrow-derived mesenchymal stromal cells (BMSCs), human dental bud stem cells and mouse clonal stromal cell lines (ST2) *in vitro* by activating the Wnt signaling pathway [19–21].

Nonetheless, the relationship between PLD and sublesional bone loss consequent to chronic SCI remains unclear. Therefore, we analyzed the therapeutic effects of PLD against sublesional bone loss in a mouse model of chronic SCI. In addition, the effects of PLD on the differentiation, activity and function of osteoblasts and osteoclasts were evaluated using cultured bone marrow cells to determine the underlying molecular mechanisms.

## MATERIALS AND METHODS

### Animal care and study design

Male C57BL/6J mice (weighing 20-25 g) were purchased from the Laboratory Animal Center of Guangzhou University of Chinese Medicine, and housed in a humidity- and temperature-controlled environment with a 12-hour light-dark cycle (8 A.M. -8 P.M.) with free access to tap water and standard pellet diet. After one week of acclimatization, the mice were randomized into the placebo (saline) or PLD-treated sham-operated and SCI groups (n = 25-27 each). At the end of the experiment, blood samples and bilateral femora and tibiae devoid of soft tissue were collected for subsequent analysis. All experimental procedures conformed to the NIH Guide for the Care and Use of Laboratory Animals and were approved by the Institutional Animal Care and USE Committee of Guangzhou University of Chinese Medicine (No. 44005800013161).

### Surgical procedures and treatment protocol

Severe thoracic SCI was induced under deep anesthesia (pentobarbital sodium, 50 mg/kg body weight, intraperitoneal injection) according to our previously published protocol [22]. Briefly, the spinal cord with dura mater was exposed by laminectomy at the T_8-9_ level under sterile conditions, and a controlled impact was made at the velocity of 0.6 m/s and depth of 0.6 mm for 90 ms. Bladder was cleared manually twice daily after the operation until spontaneous urination was restored. The sham-operated mice underwent laminectomy with no contusion injury. Daily intramuscular penicillin injections were given for the first 5 days after surgery to prevent wound infection. Eight weeks following injury, the mice were given 40 mg/kg PLD (PubChem CID: 71311798, suspension in 0.5% CMC-Na) or 0.9% saline daily by oral gavage for another consecutive 8 weeks. The duration of treatment was determined by the fact that chronic SCI-induced bone loss is considerably more severe compared to osteoporosis caused by aging, low calcium diet, menopause and ovariectomy.

### Measurement of calcium (Ca^2+^), osteocalcin (OCN) and C-terminal telopeptide of type I collagen (CTX-1) levels

Blood samples collected at termination were centrifuged at 2000 rpm for 10 min at 4° C, and the serum was separated and then kept frozen at -80° C until analysis. Serum concentration of Ca^2+^ was measured spectrophotometrically using an autoanalyzer (Hitachi, Japan). Serum concentration of OCN (a specific product of the osteoblast) was measured using a mouse OCN immunoassay kit (Jiancheng Bioengineering Institute, China), and serum CTX-1 level was evaluated using a RatLaps™ ELISA kit from BioVendor (Czech Republic), by following the manufacturer’s directions.

### Measurement of urinary deoxypyridinoline (DPD) levels

Urinary DPD (the breakdown product of collagen during bone resorption), which reflects systemic bone resorption, is considered useful for assessing the effects of osteoporosis treatment. DPD excretion was quantified by using an ELISA kit (Xinqidi Biological Technology, China), and the results were corrected for the urinary creatinine concentration, which were determined with a commercially available kit (Western Bio-Tech, China).

### Dual-energy X-ray absorptiometry (DEXA)

The femurs and tibiae were removed from each animal and soft tissues (as cartilage, tendon and ligament) were cleaned. Areal BMD were measured by DEXA (Hologic, USA) using the small-animal program. Samples were placed on an acrylic platform of uniform thickness to obtain the BMD of distal femurs, proximal tibiae and spine (L3-L5). The coefficient of variation was 1% for BMD.

### Micro-CT (μCT) analysis and mechanical testing

**Figure 2:**
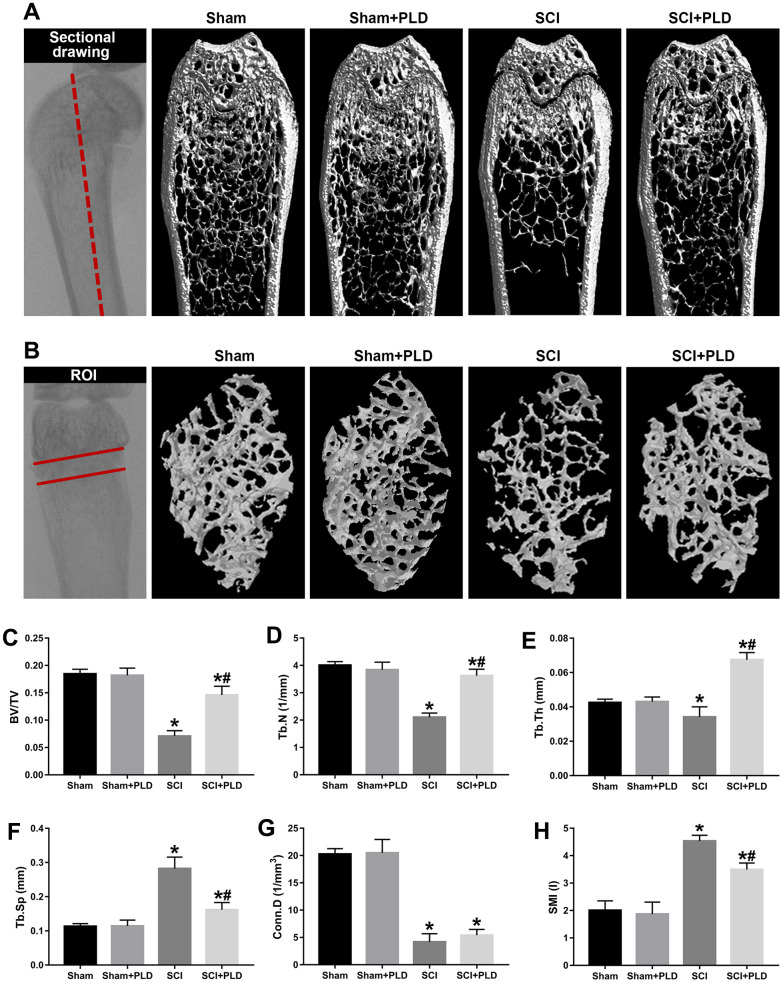
**Effects of PLD on trabecular bone structure of the distal femur.** (**A**) The schematic diagram of the coronal section of femur and representative 3D reconstructed coronal images of cancellous bone in the distal femur of each group. (**B**) The schematic diagram of the ROI of distal femur and the representative μCT 3D-images of cancellous bone within the ROI. Plots of the structural parameters: (**C**) BV/TV, (**D**) Tb.N, (**E**) Tb.Th, (**F**) Tb.Sp, (**G**) Conn.D, and (**H**) SMI. Data are expressed as mean ± S.D.; n=4 to 5 per group; *P < 0.05, vs. the Sham group; #*P* < 0.05, vs. the SCI group.

**Figure 3:**
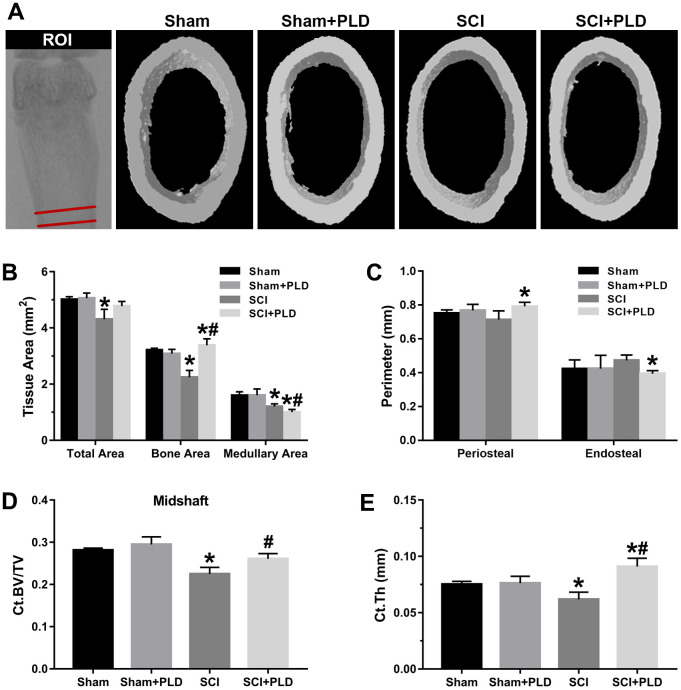
**Effects of PLD on the cortical bone structure of femur midshaft.** (**A**) The schematic diagram of the ROI of femoral midshaft and typical μCT images of cortical microarchitecture are displayed. Measurements are shown for (**B**) total, bone, and medullary tissue area, (**C**) periosteal and endosteal perimeters, (**D**) Ct.BV/TV and (**E**) Ct.Th. Data are expressed as mean ± S.D.; n=4 to 5 per group; *P < 0.05, vs. the Sham group; #*P* < 0.05, vs. the SCI group.

The morphometry of the femoral samples was measured by high-resolution μCT (Bruker, Belgium). The scanner was set using the following parameters: 59 kVp energy, 100-μA intensity, and a resolution of 9μm per pixel. Images reconstruction and three-dimensional (3D) quantitative analysis were performed with the software provided by the manufacturer. The regions of interest (ROI) of femur were defined using CTAn v1.9 (Bruker) as shown in Figures 2A, 2B, 3A. The trabecular microstructural indices were calculated, including trabecular bone volume per total bone volume (BV/TV), trabecular number (Tb.N, mm^-1^), trabecular thickness (Tb.Th, mm), trabecular separation (Tb.Sp, mm), connectivity density (Conn.D, mm^-3^), and structure model index (SMI, range from 0 to 3, with 0 = plate like and 3 = rod like). To analyze cortical bone, total bone and medullary tissue area (mm^2^), the periosteal and endosteal perimeters (mm), cortical bone volume versus total bone volume (Ct.BV/TV) and cortical thickness (Ct.Th, mm) were measured. To assess mechanical strength, the femurs were subjected to a three-point bending test at the constant velocity of 3 mm/min until complete fracture using an ElectroForce biological material testing system (TA Instruments, USA) as described by Nakagaki et al. [23].

### Bone histology and immunohistochemistry (IHC) analysis

The distal end of the femurs was fixed in 10% neutral buffered formalin (NBF) for 24 h and then decalcified in 10% EDTA (pH 7.4). The samples were then dehydrated through an ethanol gradient, embedded in paraffin, and cut into 4-μm-thick sagittal-oriented sections using a ultramicrotome. The sections were stained with hematoxylin and eosin (H&E). The expression of osteoblastic markers was detected by IHC. After deparaffinization and rehydration, the tissue sections were immersed in 3% H_2_O_2_ to block endogenous peroxidase activity, blocked with normal goat serum, and incubated overnight with the primary antibodies against OCN (1:200; Abcam, USA) and OPN (1:100; Abcam, USA) at 4° C. The following day, the sections were washed and incubated with the secondary antibodies. After developing the stain with DAB solution and counterstaining with hematoxylin, the sections were observed under a microscope (Olympus, Japan).

### Von Kossa staining and tartrate-resistant acid phosphatase (TRAP) staining

The EDTA-treated sections were further analyzed by von Kossa staining and TRAP staining. The slides were stained with the von Kossa dye and continuously irradiated under a UV lamp for 4 h. After rinsing thrice with distilled water, the sections were counterstained with H&E and observed by bright field microscopy. TRAP staining was performed using a commercially available kit (Servicebio, China). Briefly, the slices were soaked in TRAP staining solution at 37° C for 20 min. The specimens were washed in tap water, counterstained with hematoxylin, and mounted. The osteoid volume (OV) and osteoid surface (OS) were measured, and the number of osteoclasts were counted using ImageJ software (NIH, USA).

### Measurement of malondialdehyde (MDA) levels

MDA levels were measured by reacting with thiobarbituric acid (TBA) using a commercially available kit (Jiancheng Bioengineering Institute, China). Briefly, blood samples and bone homogenates were centrifuged to collect the supernatants, which were further processed according to the manufacturer’s instructions. The absorbance was measured at 532 nm using a microplate reader. The content of MDA in the serum and femur were calculated as nM and μM/mg protein respectively.

### Measurement of 25-hydroxyvitamin D [25(OH)D] levels

Serum level of vitamin D metabolite 25(OH)D was measured with a commercially available kit (R&D System, USA) following the manufacturer’s instruction manual. The intra-assay and inter-assay precision for the ELISA were less than 10% and 15% for 25(OH)D.

### Western blotting

Frozen left distal femurs were pulverized in mortar and homogenized in ice-cold IP lysis buffer (Beyotime, China) supplemented with 1mM PMSF. Samples containing approximately 30 μg protein was electrophoretically separated by 12% SDS-PAGE and transferred to PVDF membranes (Millipore, USA). After blocking non-specific binding with 5% skimmed milk in TBST for 1h at room temperature, the membranes were incubated overnight with primary antibodies at 4° C. The membranes were then washed thrice with TBST (10 min each time), and incubated with the secondary antibodies for 2 h. After extensive washing, the bands were visualized using an ECL detection kit (Beyotime) and densitometrically quantified with Image Lab version 2.1 (Bio-Rad). GAPDH was used as an internal control.

### Culture and differentiation of bone marrow progenitors

Procedures for study of osteoblast and osteoclast formation were followed the methods previously described [24]. Briefly, the bone marrow cells were extracted from the femurs and tibiae under sterile conditions. To initiate osteoblast differentiation, the BMSCs were cultured in α-MEM supplemented with 10% fetal bovine serum (Gibco, USA) and 1 mM ascorbic acid-2-phosphate, and the medium was replaced every two days. The formation of osteoblastic lineage cells was determined by staining the colonies with alkaline phosphatase (ALP) on day 10, and the number of ALP-positive colonies (colony-forming unit–fibroblastic, CFU-F) were counted. The cells producing mineralized bone matrix were stained with the von Kossa dye on day 28, the number of positive colonies (CFU–osteoblastic, CFU-OB) was counted. In addition, ALP activity assay and Alizarin red staining were performed on day 7 and day 14 of induction, respectively, as previously described [25]. The BMSCs were differentiated into osteoclasts by culturing in α-MEM supplemented with human macrophage colony-stimulating factor (M-CSF) for 2 days. Non-adherents were collected, purified by Ficoll-Plus, and incubated for another 5 days in *α*-MEM supplemented with M-CSF (30 ng/mL) and RANKL (60 ng/mL). Osteoclasts were identified by staining for TRAP. Total RNA was also extracted from the induced cells for RT-qPCR.

### Quantitative real-time PCR analysis

The right distal femurs were snap frozen in liquid nitrogen and ground into a powder. Total RNA was extracted using Trizol reagent (Invitrogen, USA) according to the manufacturer’s guidelines, and 50 ng template was reverse-transcribed into cDNA using the PrimeScript™ RT Master Mix Kit (Takara, Japan). Quantitative real-time PCR was performed using a SYBR Green PCR Mix on the CFX96™ Real-Time PCR Detection System (Bio-Rad, USA) as previously described [22]. Each sample was run and analyzed in triplicate using the 2^-ΔΔCt^ method, and expressions of Runt-related transcription factor 2 (RUNX2), OCN, ALP, osteoprotegerin (OPG), Receptor-activator of Nuclear Factor κ-B Ligand (RANKL), calcitonin receptor (CTR), TRAP, sclerostin (SOST), wingless-related MMTV integration site 3A (Wnt3a), lipoprotein receptor-related protein 5 (Lrp5) and ctnnb1 were normalized to *β*-actin. The sequences of primers used for amplification of target genes were listed in Table 1.

**Table 1:** Sequences of primers used in the RT-qPCR.

**Primer**	**Gene ID**		**Sequence of primers (5’-3’)**	**Product size/bp**
RUNX2	NM_001146038.2	Forward	CAGTCCATGCAGGAATATTTAAGGC	142
		Reverse	TCCCAAAAGAAGCTTTGCTGA	
OCN	NM_007541.3	Forward	GCAGAACAGACAAGTCCCACAC	83
		Reverse	GTCAGCAGAGTGAGCAGAAAGAT	
ALP	NM_007431.3	Forward	CTTCATAAGCAGGCGGGGGA	180
		Reverse	GAGCCCAGATGGTGGGAAGA	
OPG	NM_008764.3	Forward	TTAACCCCGGAGTGTCCCAAA	109
		Reverse	CAGGAAGTATGCCCTGCCTTT	
RANKL	NM_011613.3	Forward	GAGCACGAAAAACTGGTCGG	111
		Reverse	GGGTTGGACACCTGAATGCT	
CTR	NM_001042725.1	Forward	GCAGGCACTGCTAAGGAGA	166
		Reverse	GGTGTTCTCAGGAACGCAGA	
TRAP	NM_011616.2	Forward	ATGGGAAACAGCTGACGGTT	99
		Reverse	TGAATGGGCGTTGACTCGAA	
SOST	NM_024449.6	Forward	GGCAAGCCTTCAGGAATGATG	188
		Reverse	TGTCAGGAAGCGGGTGTAGT	
Wnt3a	NM_009522.2	Forward	TACTACGAGGCCTCACCCAA	103
		Reverse	ACCCATCTATGCCATGCGAG	
Lrp5	NM_008513.3	Forward	GGCCAGTGTGTCCTCATCAA	194
		Reverse	ACACGCTGGCAGACAAAGTA	
Ctnnb1	NM_007614.3	Forward	CGCCGCTTATAAATCGCTCC	81
		Reverse	TTCACAGGACACGAGCTGAC	
*β*-Actin	NM_007393.5	Forward	TGAGCTGCGTTTTACACCCT	198
		Reverse	GCCTTCACCGTTCCAGTTTT	

### Statistical analysis

All data were analyzed statistically using SPSS 24.0 software (SPSS Inc., USA), and expressed as mean ± S.D. Statistical comparisons among groups were made by one-way analysis of variance (ANOVA) or unpaired Student’s *t*-test. Graphs were generated by GraphPad Prism 6.0 software (GraphPad Software, USA). A probability value of *P* less than 0.05 was considered statistically significant.

### Data availability statement

The datasets used to support the findings of this study are available from the corresponding author upon request.

## RESULTS

### Effects of PLD treatment on the general indices of chronic SCI

As shown in Table 2, although there was no significant difference in the initial body weight among the groups, mice with SCI weighed slightly less compared to the sham-operated mice at the end of the experiment. The serum levels of Ca^2+^ and OCN in the SCI group were significantly lower compared to that in the blank control group. In addition, SCI mice exhibited enhanced bone resorption, as indicated by the marked increase in serum CTX-1 and urine DPD levels. PLD treatment significantly increased serum OCN levels, decreased serum CTX-1 and urinary DPD levels, and slightly improved the body weight of the mice with chronic SCI. However, PLD treatment had no significant effect on the serum Ca^2+^ levels of the SCI model, or on any of the above parameters in the sham-operated control mice.

**Table 2:** Effects of treatment with polydatin on general data of chronic SCI mice.

	**Sham**	**Sham+PLD**	**SCI**	**SCI+PLD**
Body mass (g)				
Before SCI	22.0±1.1	21.8±0.9	21.5±1.2	21.5±1.4
At Sacrifice	30.7±1.2	30.4±1.4	26.8±1.9^a^	28.6±1.5
Serum Ca^2+^ (mg/dL)	10.05±0.36	10.46±0.91	7.90±1.29^a^	8.25±0.71^a^
Serum OCN (ng/mL)	5.22±0.23	5.14±0.19	3.80±0.44^a^	4.64±0.32^ab^
Serum CTX-1 (ng/mL)	501±28	505±26	799±36^a^	609±43^ab^
Urine DPD (nM/mM creatine)	2.5±0.3	2.5±0.4	8.0±0.7^a^	4.2±0.5^ab^

Values are represented as mean ± SD.

Sham, sham-operated; SCI, chronic spinal cord injury; PLD, polydatin; OCN, osteocalcin; CTX-1, type I collagen cross-linked C-terminal peptide; DPD, deoxypyridinoline.

*n*=10–12 in each group.

^a^*P*<0.05 versus the sham-operated mice.

^b^*P*<0.05 versus the SCI mice.

### PLD reversed chronic SCI-induced bone loss

Mice with SCI had 34.8%, 35.1%, and 8.3% lower BMD compared to the sham-operated mice in the distal femoral metaphysis (DFM), proximal tibial metaphysis (PTM) and lumbar vertebrae, respectively. The loss of BMD in both lower limbs was similar (data not shown). Eight weeks after PLD administration, the BMD of the DFM and PTM were restored to that of the sham-operated group (Figure 1C, 1D). It is worth noting that the BMD of the lumbar spine was slightly greater for PLD-treated SCI mice compared to that of the sham-operated mice (Figure 1E). Chronic SCI also decreased the BV/TV, Tb.N and Tb.Th, and increased the Tb.Sp at this site compared to the sham-operated controls (Figure 2C–2F). In addition, trabecular Conn.D was significantly reduced after SCI, and associated with the transition from plate-like to rod-like structures (Figure 2G, 2H). Treatment of SCI mice with PLD significantly increased the BV/TV ratio, primarily by increasing Tb.Th (+ 97.6% vs. SCI) and reducing Tb.Sp (- 42.7% vs. SCI), with a lesser effect on Tb.N (Figure 2C–2F). Furthermore, a slight (statistically non-significant) increase in Conn.D was also noted in chronic SCI mice due to SMI reduction (Figure 2G, 2H).

**Figure 4:**
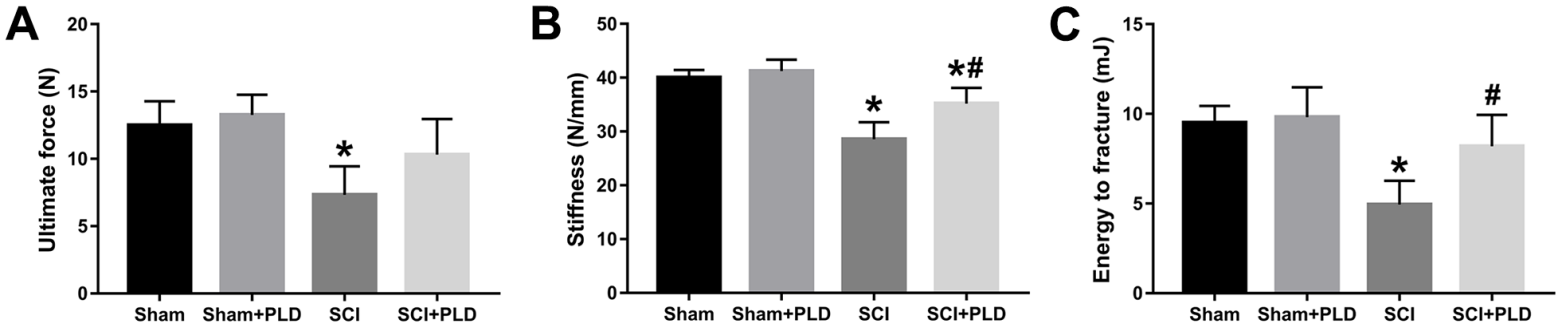
**Effects of treatment with PLD on biomechanical parameters of mouse femurs.** (**A**) Ultimate force, (**B**) stiffness, and (**C**) energy to fracture of the femurs among the four groups of mice. Data are expressed as mean ± S.D.; n=4 to 5 per group; *P < 0.05, vs. the Sham group; #*P* < 0.05, vs. the SCI group.

High-resolution μCT was also used to evaluate the effect of PLD on the cortical bone structure in the femur midshaft. The cortical BMD of all 4 groups was similar 16 weeks after surgery (data not shown). However, the SCI mice had thinner bones and reduced total area, bone area, and medullary area compared to the sham-operated group (Figure 3B), resulting in a slight decrease in Ct.Th (Figure 3E). Interestingly, both Ct.BV/TV (+ 16.1% vs. SCI; Figure 3D) and Ct.Th (+ 42.2% vs. SCI; Figure 3E) were significantly increased in the PLD-treated SCI animals due to a moderate increase in periosteal perimeter (+ 10.9% vs. sham; Figure 3C), as well as significant reductions in medullary area (- 16.6% vs. sham; Figure 3B) and endosteal perimeter (- 16.5% vs. SCI; Figure 3C). The femoral diaphysis of the SCI mice had significantly lower ultimate force and stiffness, and required less energy to fracture compared to that of the sham-operated group (Figure 4). PLD treatment significantly improved the mechanical properties of the bones of SCI mice. However, 8 weeks of PLD treatment had no significant effect on the bone geometry, microstructure and mechanical properties of sham-operated mice.

### PLD regulated bone formation and bone resorption in chronic SCI mice

**Figure 5:**
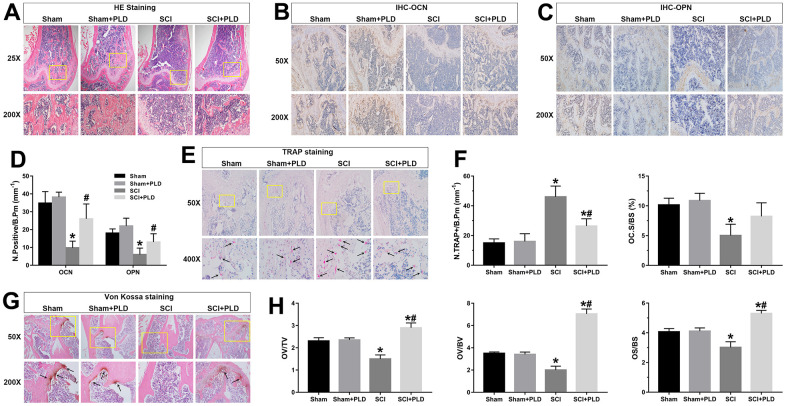
**Effects of PLD on bone morphology, bone formation, calcium deposition and bone resorption.** (**A**) Representative photomicrographs of H&E-stained sections. IHC analysis of (**B**) OCN and (**C**) OPN protein expression in distal femurs. (**D**) Number of OCN^+^ and OPN^+^ osteoblasts on the bone surface. (**E**) Representative images of TRAP-stained distal femoral sections. Arrows indicate TRAP^+^ cells. (**F**) Histo-morphometric quantification of osteoclast number per bone perimeter (N.TRAP+/B.Pm), and normal osteoclasts surface per bone surface (OC.S/BS). (**G**) Representative images of von Kossa-stained mouse femoral trabecular bone sections. Arrows indicate the presence of mineralized bone. (**H**) Quantitative analysis of osteoid volume versus total volume (OV/TV), osteoid volume versus bone volume (OV/BV), and osteoid surface versus bone surface (OS/BS). Measurements were presented as mean ± S.D.; n=6 to 7 per group; *P < 0.05, vs. the Sham group; #*P* < 0.05, vs. the SCI group.

We next analyzed the effect of PLD treatment on bone formation and bone resorption in terms of histological changes, osteoblastogenesis and osteoclastogenesis markers, and calcium deposition in the femurs. H&E staining of DFM revealed smaller, thinner trabeculae with more lipid droplets and microfractures in the SCI group relative to the sham-operated controls. In contrast, PLD treatment largely attenuated the SCI-induced histo-morphological damage (Figure 5A).

As shown in Figure 5B–5D, the *in-situ* expression of OCN and OPN was markedly decreased in the vehicle-treated SCI animals, suggesting inhibition of osteoblastic differentiation. PLD treatment improved bone formation in DFM, which was indicated by an increased number of OCN-positive mature osteoblasts and OPN-positive pre-osteoblasts on the bone surfaces. Furthermore, von Kossa/Tetrachrome staining revealed a marginal reduction in osteoid volume (OV/TV and OV/BV) and osteoid surface (OS/BS) in the SCI group compared to the sham-operated group (Figure 5G, 5H), which were substantially elevated after PLD treatment to levels higher than that of the sham-operated mice.

The number of TRAP^+^ osteoblasts (N.TRAP^+^/B.Pm) in the SCI group was 46 ± 7.28 per bone perimeter (mm^-1^), which was significantly higher compared to 15 ± 2.73/mm^-1^ in the sham-operated group (Figure 5E, 5F). After PLD administration, the number of N.TRAP^+^/B.Pm cells decreased significantly. Moreover, the normal osteoclasts surface per bone surface (OC.S/BS) of vehicle-treated and PLD-treated SCI mice showed an opposite trend. PLD treatment had not significant effect on the number of osteoblasts and osteoclasts in the sham-operated animals. Taken together, PLD enhanced bone formation and suppressed bone resorption in chronic SCI mice.

### PLD abated oxidative stress and attenuated MMP activity in chronic SCI mice

**Figure 6:**
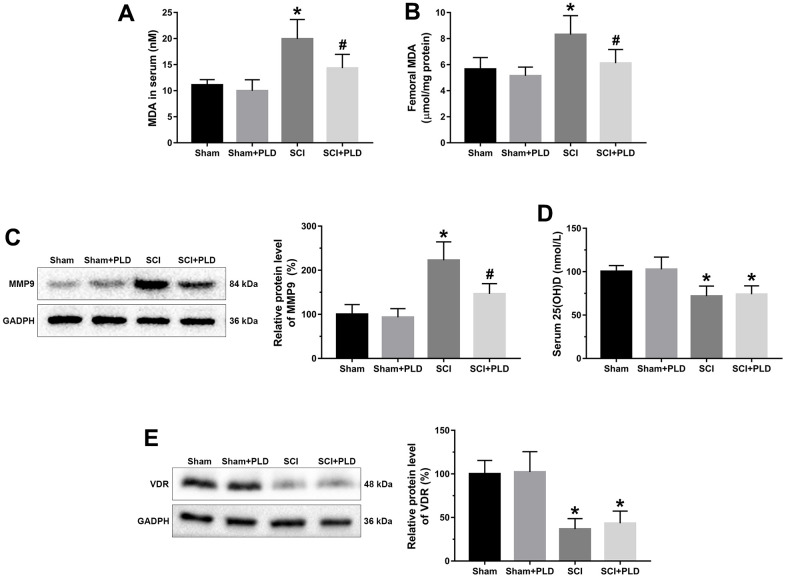
**Effects of treatment with PLD on oxidative stress and vitamin D deficiency in serum and femurs of mice.** MDA levels in serum (**A**) and femurs (**B**) were determined using a kit. (**C**) The expression level of MMP-9 in femoral tissues was detected by immunoblot. (**D**) Serum 25(OH)D levels were determined by radioimmune assay. (**E**) Femoral protein expression of VDR were determined by Western blotting and quantification analysis were shown. The protein levels of MMP-9 and VDR were adjusted as relative values to GAPDH. Data are expressed as mean ± S.D.; n=5 to 7 per group; *P < 0.05, vs. the Sham group; #*P* < 0.05, vs. the SCI group.

MDA level is an indicator of lipid peroxidation and oxidative stress levels. Compared to the sham-operated mice, the serum and femoral MDA content were higher in the chronic SCI mice (Figure 6A, 6B). PLD supplementation significantly decreased serum and femoral MDA levels, which is consistent with its anti-oxidant effects. Induction of SCI also activated femoral MMP9, which plays a key role in bone resorption (Figure 6C), compared to the control group. Treatment of chronic SCI mice with PLD significantly decreased protein expression of MMP9 in the femur.

### PLD had no effect on 25(OH)D and VDR in chronic SCI mice

We assessed the effect of PLD therapy on the reversal of vitamin D deficiency by detecting serum 25(OH)D levels and VDR protein expression in the distal femurs. Although the serum 25(OH)D and VDR protein expression levels in SCI group were significantly lower than that in the sham-operated group, PLD treatment had no effect on these parameters (Figure 6D, 6E).

### PLD enhanced osteoblastogenesis in mice with chronic SCI

**Figure 7:**
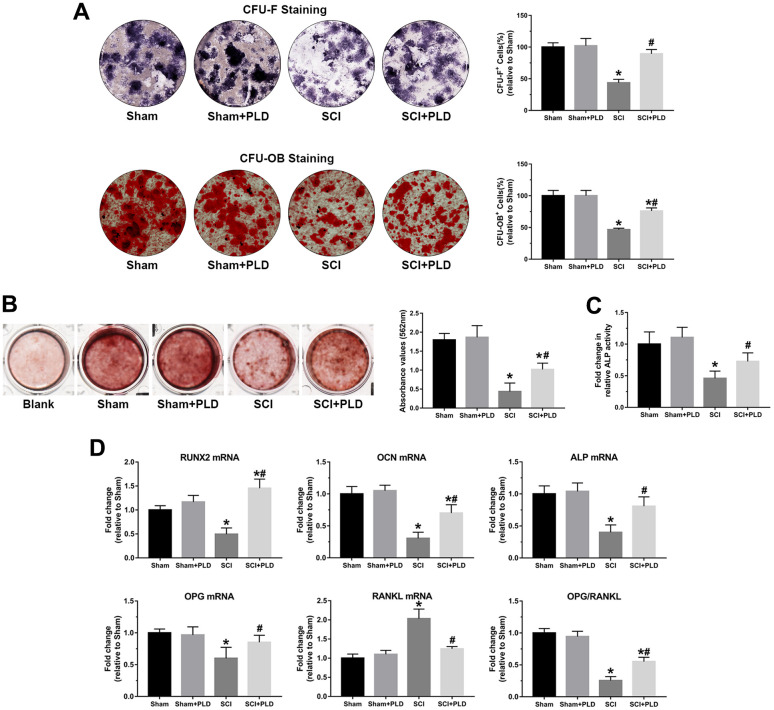
**PLD promoted osteoblastogenesis in bone marrow progenitor cells generated from chronic SCI mice.** (**A**) The number of osteoblast-like cells (CFU-F) and the number of mineralized nodules (CFU-OB) in BMSC cultures were identified by ALP staining and von Kossa staining, respectively. (**B**) Alizarin red staining was used to evaluate BMSC calcification in the presence or absence of OIM of in groups. (**C**) Quantitative analysis of the ALP activity assay. (**D**) RUNX2, OCN, ALP, OPG and RANKL mRNA levels, and OPG/RANKL ratio in the osteoblast-like cells. mRNA levels were determined by qRT-PCR analysis. Data are expressed as mean ± S.D.; n=4 per group; *P < 0.05, vs. the Sham group; #*P* < 0.05, vs. the SCI group.

At 16 weeks after SCI induction, a marked reduction was observed in the number of ALP^+^ osteoblast-like cells (CFU-F), an indicator of the differentiation of bone marrow precursors into the osteoblast lineage (Figure 7A). Similarly, the number of mineralized nodules (CFU-OB) was also significantly reduced in the SCI group (Figure 7A). PLD treatment restored osteoblast differentiation in the affected bones. As shown in Figure 7B, PLD promoted BMSC calcification in animals with chronic SCI, as evidenced by Alizarin red staining. This result was also consistent with the outcome of the quantitative analysis of the ALP activity assay (Figure 7C).

Total RNA was isolated from osteoblast-like cells derived from bone marrow stromal precursors *in vitro* to detect the transcript levels of osteogenic differentiation markers. Compared with the sham-operated group, the chronic SCI group showed a 50%, 69% and 60% reduction in the relative expression of RUNX2, OCN and ALP, respectively (Figure 7D). PLD treatment significantly increased all transcripts to levels similar to that of the sham-operated group. OPG mRNA expression was reduced by approximately 40% in the chronic SCI mice compared to the sham-operated controls, which was accompanied by a 2-fold elevation in RANKL mRNA expression (Figure 7D). Treatment of SCI mice with PLD significantly upregulated OPG and downregulated RANKL mRNA levels, resulting in 2-fold increase in the OPG/RANKL ratio. There was no significant difference between the two control groups in terms of the above transcripts.

### PLD inhibited osteoclastogenesis in mice with chronic SCI

**Figure 8:**
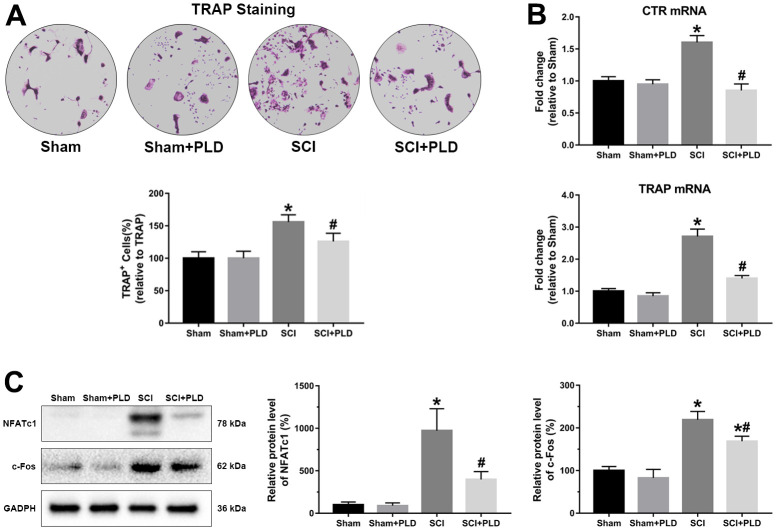
**PLD inhibited the osteoclastogenic potential of bone marrow precursors isolated from chronic SCI mice.** (**A**) The number of osteoclasts were identified by TRAP staining. (**B**) CTR and TRAP mRNA levels in cultured bone marrow progenitor cells. mRNA levels were determined by qRT-PCR analysis. (**C**) NFATc1 and c-Fos protein expression was detected by Western blotting and adjusted as relative values to GAPDH. Data are expressed as mean ± S.D.; n=4 per group; *P < 0.05, vs. the Sham group; #*P* < 0.05, vs. the SCI group.

The osteoclastic potential of bone marrow hematopoietic precursors in different groups was determined by counting the number of TRAP^+^ multinucleated cells (MNCs). Consistent with previous findings [26, 27], TRAP^+^ MNCs increased by 55% in bone marrow cell cultures from mice with chronic SCI compared to that of the sham-operated controls (Figure 8A). Administration of PLD significantly reduced the number of TRAP^+^ MNCs by 19% in animals with chronic SCI.

Consistent with this, the osteoclast differentiation markers CTR and TRAP were significantly upregulated by about 60% and 170%, respectively, in osteoclast cultures from animals with chronic SCI. After PLD treatment, the mRNA levels were decreased to levels similar to those detected in vehicle-treated or PLD-treated sham-operated animals (Figure 8B). In addition, the protein expression of osteoclast target genes (NFATc1 and c-Fos) also showed a similar trend (Figure 8C).

### PLD activated the Wnt/*β*-catenin pathway in the femurs of chronic SCI mice

**Figure 9:**
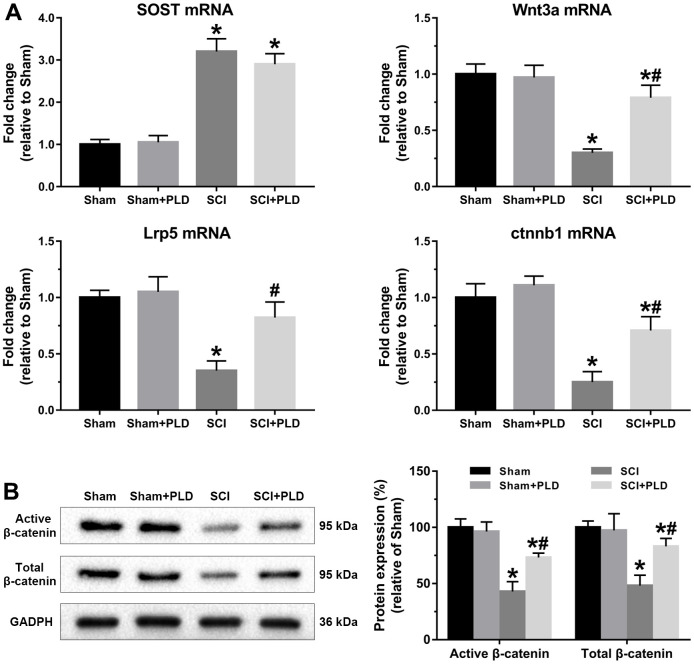
**PLD activated Wnt/*β*-catenin signaling pathway in the femurs of chronic SCI mice.** (**A**) SOST, Wnt3a, Lrp5 and ctnnb1 mRNA levels were determined by qRT-PCR method. (**B**) Immunoblot showing active *β*-catenin and total *β*-catenin levels. The protein levels of *β*-catenin were adjusted as relative values to GAPDH. Data are expressed as mean ± S.D.; n=5 to 7 per group; *P < 0.05, vs. the Sham group; #*P* < 0.05, vs. the SCI group.

Wnt/*β*-catenin signaling was evaluated by analyzing the femoral levels of SOST, Wnt3a, Lrp5, and ctnnb1 mRNAs. SOST mRNA levels in the chronic SCI mice were approximately 2-fold higher compared to that in the sham-operated group, and unaffected by PLD treatment. Compared to the sham-operated group, Wnt3a, Lrp5, and ctnnb1 mRNAs were significantly downregulated in the SCI group, and restored to varying degrees by PLD (Figure 9A). Strikingly, Lrp5 expression in the PLD-treated SCI group was similar to that of the sham-operated controls. In addition, both the active and total *β*-catenin protein levels were decreased in the femurs of chronic SCI mice and reversed after PLD treatment (Figure 9B). Taken together, PLD exerted its therapeutic effects against chronic SCI by activating the femoral Wnt/*β*-catenin signaling.

## DISCUSSION

Low bone mass, reduced BMD and deterioration of bone microarchitecture are some of the serious complications of chronic SCI, predominantly occur in the pelvis and lower extremities. Bone loss increases the risk of spontaneous low-impact fractures of the lower limbs, and exacerbates disability [28]. Sublesional skeletal deterioration can be detected radiologically as early as 6 weeks after SCI (acute phase), whereas a more significant BMD reduction is observed at 3 to 5 years post-injury (chronic phase) [29, 30]. The bone loss pattern in SCI patients differs from that commonly encountered in cases of endocrine disorders and disuse osteoporosis. Although drugs like bisphosphonates, denosumab and teriparatide can partially prevent osteoporosis in the acute post-injury phase [31–33], there are no specific clinical practice guidelines for preventing/managing bone loss during chronic SCI [27]. In this study, we have shown for the first time that PLD can mitigate chronic SCI-induced sublesional osteoporosis in mice.

SCI patients often exhibit significant reduction in trabecular bone mass, especially at the DFM and PTM. Consistent with this, we detected loss of trabecular bone mass and impaired skeletal microarchitecture in the mouse model 16 weeks after injury. Previous studies have also shown severe cancellous bone deterioration in animal models with chronic SCI [27], indicative of compromised structural integrity and abridged mechanical strength. PLD administration almost restored the trabecular bone volume, number and separation in chronic SCI animals to the normal levels, and the trabecular bone thickness was even significantly greater than that observed in sham-operated controls. Moreover, SCI-induced deterioration of trabecular bone connectivity was also slightly improved by PLD, as indicated by a denser structure with more “plate-like” trabeculae and highly interconnected forms, which increases resistance to higher loads. A previous preclinical study reported that motor-incomplete SCI had no effect on the cortical area and thickness of the hindlimb long bones [34]. However, in our chronic spinal cord contusion model, bone tissue area was markedly reduced and cortical thinning was also detected, which positively correlated with peak load of the femoral diaphysis [30]. PLD significantly reduced endosteal perimeter and medullary area, and increased midshaft femur cortical volume and cortical thickness, which translated into greater mechanical integrity and resistance to fracture.

SCI-induced sublesional osteoporosis can be attributed to insufficient reactive bone formation and increasing bone resorption [35]. Serum OCN is a marker of bone formation [36], and was significantly lower in SCI mice. In contrast, two specific bone resorption biomarkers, urine DPD and serum CTX-1, were increased in SCI mice [37]. Eight weeks of PLD administration restored the above markers to near normal levels, indicating its role in regulating the balance between bone formation and bone resorption. Consistent with this, PLD attenuated the morphological damage in the trabecular bones of SCI mice. It also enhanced the rate of bone formation by increasing the number of OCN-positive mature osteoblasts and OPN-positive pre-osteoblasts on the bone surface, and inhibiting osteoclast generation. Therefore, PLD rescued chronic SCI mice from sublesional bone loss by enhancing bone formation and suppressing bone resorption.

Osteoblast cultures treated with PLD showed an increased number of calcified nodules and CFUs, indicating that PLD can enhance the osteogenic potential of bone marrow progenitor cells. Furthermore, PLD significantly increased the expression of genes promoting osteoblast differentiation *ex vivo*. These findings are consistent with a recent study which showed that PLD enhanced the proliferation of hBMSCs and increased calcium deposition by upregulating osteogenic genes [20]. The balance between bone formation and resorption is modulated by the RANKL/RANK/OPG signaling pathway. RANKL is a membrane-bound factor expressed by osteoclastogenic cells, and plays an indispensable role in maintaining of bone homeostasis. It binds to and activates its cognate receptor RANK, which promotes osteoclast recruitment and activity, eventually leading to bone resorption [38, 39]. OPG is produced by osteoblasts/stromal cells and acts as an antiresorptive agent that inhibits osteoclast maturation by competitively binding to RANKL, thereby blocking RANKL-RANK interaction [39]. Consistent with the data from other models of bone resorption, we found that RANKL mRNA was markedly upregulated in osteoblast-like cells induced from BMSCs of SCI mice [40]. PLD abolished the elevated osteoclast precursor population (TRAP^+^ MNCs) after chronic SCI, most likely by upregulating the OPG/RANKL axis in osteoblasts.

Vitamin D deficiency is an important risk factor contributing to declining BMD and increased risk of fragility fractures in patients with SCI. In a prospective randomized controlled trial, administration of vitamin D analog restored bones loss in the lower limbs of chronic SCI patients and decreased urinary excretion of N-telopeptide (a marker of bone resorption), but had no significant effect on the levels of osteogenic genes [11]. However, PLD did not alter serum 25(OH)D and femoral VDR protein levels in the present study. VDR is a member of the steroid/retinoid receptor superfamily of nuclear factors that mediates the pleiotropic biological actions of 25(OH)D, thereby affecting calcium metabolism and bone homeostasis [41]. Thus, the therapeutic effect of PLD on SCI-induced bone loss was independent of vitamin D.

Oxidative stress is a hallmark of secondary injuries associated with SCI. In our study, increased serum and femoral MDA levels were observed in the SCI mice, suggesting that oxidative stress is a factor in the progression of sublesional bone loss following chronic SCI. Sun et al. recently reported that oxidative stress may be related to the development of disuse-related osteoporosis in rats with hindlimb suspension [42]. In addition, reactive oxygen species (ROS), a byproduct of aerobic respiration and metabolism, is generated following RANKL stimulation and modulates RANKL-mediated activation of Akt, MAPKs and NF-κB pathways [43, 44], mainly contributing to early osteoclast development and later NFATc1 and c-Fos activation [45]. NFATc1, a well-known transcription factor in osteoclast differentiation and proliferation, is considered to be a key RANKL-induced signal transducer [46]. Furthermore, osteoclast specific genes, including c-Fos, Atp6v0d2, Ctsk and MMP9, are all directly regulated by NFATc1 [47]. Thus, ROS plays a key role in RANKL-induced osteoclastogenesis [6]. PLD is a dual-function anti-oxidant that directly scavenges ROS and also upregulates anti-oxidant and cytoprotective genes. We have previously reported that PLD protected BMSCs against H_2_O_2_-induced oxidative damage and cell death [48], and reversed oxygen–glucose-deprivation/reperfusion-induced neuronal and mitochondrial damage by mitigating oxidative stress [49]. In this study, PLD decreased MDA levels in the serum and femur of chronic SCI animals, while inhibiting the expression of NFATc1 and c-Fos. We also detected high MMP9 activity in the femurs of SCI mice, which is consistent with a previous report [50]. MMP-dependent matrix degradation is crucial for bone resorption and destruction, and MMP inhibitors increased the expression of type I collagen and the BMD in ovariectomized aged rats [7]. High levels of ROS also promote collagen degradation by stimulating MMP activity [8], and PLD significantly alleviated the symptoms of collagen-induced arthritis in mice by reducing MMP9 activity [51].

The SOST gene was upregulated in the distal femurs of mice following chronic SCI, which is consistent with reports correlating elevated SOST levels with bone loss in chronic SCI patients [52]. The Wnt/*β*-catenin pathway was also repressed in the SCI mice, evidenced by the downregulation of Wnt3a, Lrp5 and ctnnb1, suggesting that sclerostin/Wnt/*β*-catenin is likely involved in regional osteoporosis after SCI. Canonical *β*-catenin-dependent Wnt signaling is initiated when a Wnt signaling proteins (such as Wnt3a) binds to and activates a receptor complex of frizzled receptor Lrp5 [53]. Wnt/*β*-catenin signaling stimulates bone formation by promoting directional osteogenic differentiation of BMSCs, and inhibits bone resorption by upregulating OPG production and reducing osteoclastogenesis [54]. The SOST-encoded protein sclerostin inhibits osteoblastogenesis by blocking the Wnt/*β*-catenin pathway through competitive binding with Lrp5, and modulates skeletal response to mechanical unloading [26, 34, 55]. Sclerostin also functions as the main mediator of bone loss after SCI [26, 34], and SOST-deficient mice were resistant to cancellous hindlimbs, cortical bone deficits osteoporosis in the sublesional regions of the lower extremities after SCI [24]. PLD can mediate the anabolic actions of canonical Wnt/*β*-catenin signaling in BMSCs and osteosarcoma cancer cells [20, 56]. We found that PLD had no marked effect on SOST expression, but restored Wnt/*β*-catenin signaling. Liposomal Wnt3a enhances cell survival and increases the osteogenic capacity of aged bone grafts [57]. However, increased in lipoxygenase-mediated lipid oxidation can lead to oxidative stress in the osteoblasts, thereby diminishing the pro-osteogenic effect induced by Wnt/*β*-catenin activation [58]. Thus, we can surmise that the regulatory effect of PLD on Wnt signaling may be based on its anti-oxidant action rather than up-regulation of SOST.

There are several limitations in our study that ought to be considered. First, although weight loss is a major risk factor of post-SCI osteoporosis, neural injury also plays a major role [28, 30]. In a previous study, PLD significantly attenuated neuronal damage and partially promoted the recovery of hindlimb locomotor function after SCI [22, 49]. Therefore, it is still unclear whether PLD improved sublesional osteoporosis by directly affecting the bone, or indirectly through its neuroprotective effects, or through a combination of both. Secondly, the cytokines and biomarkers of bone turnover in the sham-operated mice were completely unresponsive to PLD. Based on previous studies, we administrated PLD at the dose of 40 mg/kg/day, which may explain the lack of any potential impact on the bones of healthy animals. Therefore, further studies are needed with higher drug dosage, different time points, and on a larger sample size.

In conclusion, PLD mitigated severe sublesional bone loss after chronic SCI by reducing oxidative stress, inhibiting MMP activity, and activating the Wnt/*β*-catenin pathway. Therefore, PLD can be considered as a potential treatment strategy for SCI-induced bone loss, and warrants clinical testing.
